# Comprehensive Regulation of Liquid–Liquid Phase Separation of Polypeptides

**DOI:** 10.3390/molecules28186707

**Published:** 2023-09-20

**Authors:** Yanwei Wang, Dongxin Xiang, Siyuan Chen, Guangcan Yang

**Affiliations:** Department of Physics, Wenzhou University, Wenzhou 325035, China; wangyw@wzu.edu.cn (Y.W.); 20451025001@stu.wzu.edu.cn (D.X.); 21451026003@stu.wzu.edu.cn (S.C.)

**Keywords:** liquid-liquid phase separation, polypeptides, electrostatic interaction, hydrophobicity

## Abstract

The elucidation of the molecular driving forces responsible for Liquid–liquid Phase Separation (LLPS) of proteins and nucleic acids within living cells is crucial for understanding its biological functions and its role in related diseases. In the present study, we investigated the regulation of LLPS in a series of polypeptides with repetitive proline and arginine (PR) sequences by modifying their length and the salt concentration in the solution. Our findings indicate that higher salt concentrations are necessary for LLPS of repetitive PR peptides longer than eight PRs, which emerges as a threshold value. To pinpoint the molecular forces driving the LLPS in peptides, we sequentially introduced various concentrations of hydrophobic disruptors, such as 1,6-hexanediol, and electrostatic regulators, such as ethyl alcohol and 6-Aminocaproic acid. We further modulated the electrostatic interaction by introducing ethyl alcohol and 6-Aminocaproic acid to alter the dielectric constant of the solution. The inclusion of ethyl alcohol intensified the electrostatic interaction between arginine molecules, facilitating LLPS of PR15, while 6-Aminocaproic acid yielded the reverse effect. We deduced that the phase separation in peptide systems is conjointly driven by hydrophobicity and electrostatic interactions. These insights can guide the regulation of LLPS in other peptide and protein systems, and could be pivotal in addressing abnormal aggregations of proteins and nucleic acids.

## 1. Introduction

Liquid–Liquid Phase Separation (LLPS) is instrumental in forming membraneless organelles within living cells, stemming from protein and nucleic acid interactions [1,2]. Moreover, metastable LLPS in protein solutions represents a key biophysical phenomenon, potentially shedding light on the intricacies of biological structure genesis [3,4,5,6,7]. Recognized as an essential organizing principle, LLPS facilitates the condensation of proteins and other biomolecules into liquid droplets, which underpin the creation of membrane-less subcellular domains [8]. The orchestration of LLPS in cells plays a part in various biological undertakings—from chromatin restructuring to noise buffering and sensing. Furthermore, it is implicated in numerous pathologies, including neurodegenerative disorders and cancer [2]. Data suggest that LLPS is influenced by elements like ionic intensity, pH, ambient temperature, and the nature of salts and added substances [9]. Prior experimental and theoretical works have dissected the mechanism of liquid–liquid separation from diverse perspectives [10,11,12].

LLPS exemplifies a thermodynamic phenomenon where, primarily through weak interactions, the biomolecules segregate into dilute and concentrated phases to minimize their free energy [13,14,15]. Balancing the entropy and enthalpy forces in this process presents an intricate challenge, with charge–charge, cation-π, dipole–dipole, and π–π molecular interactions at play [2]. Several determinants, including component concentration, chemical makeup, temperature, and the presence of external molecules, can steer LLPS in solutions [16,17]. Within cells, LLPS regulation hinges on modulating protein–protein liaisons. Proteins intrinsic to LLPS usually possess distinct domains like coiled-coil regions, facilitating complex formation via protein interplays. Post-translational tweaks, such as phosphorylation, can recalibrate these interactions by altering protein conformation, thereby influencing its LLPS role [18]. In essence, orchestrating LLPS within cells is multifaceted, driven by a plethora of variables. Nonetheless, unravelling the LLPS regulatory mechanisms is pivotal for demystifying cellular phenomena, ranging from signaling to gene modulation and disease manifestation.

In our research, we probe the LLPS influenced by the polymerization length of a peptide, embodying repetitive proline and arginine sequences, and by the dielectric traits of salt solutions. This specific peptide sequence, notorious for its cellular toxicity, is encoded by the C9ORF72 gene [19,20], intrinsically linked to amyotrophic lateral sclerosis (ALS). Presently, ALS poses a grave challenge to humanity, with its onset closely tied to anomalous aggregations ensuing from the liquid phase segregation of affiliated proteins [21,22,23]. We discern that for the repetitive proline and arginine (PR) peptides, a more elevated salt concentration becomes essential for LLPS as its length surpasses a certain threshold. Examining the repetitive PR series’ regulation may provide insights into ALS’s underlying mechanisms and inspire potential therapeutic strategies.

## 2. Results and Discussion

### 2.1. Effect of Salinity on LLPS in the PR Peptide Series

**Figure 1:**
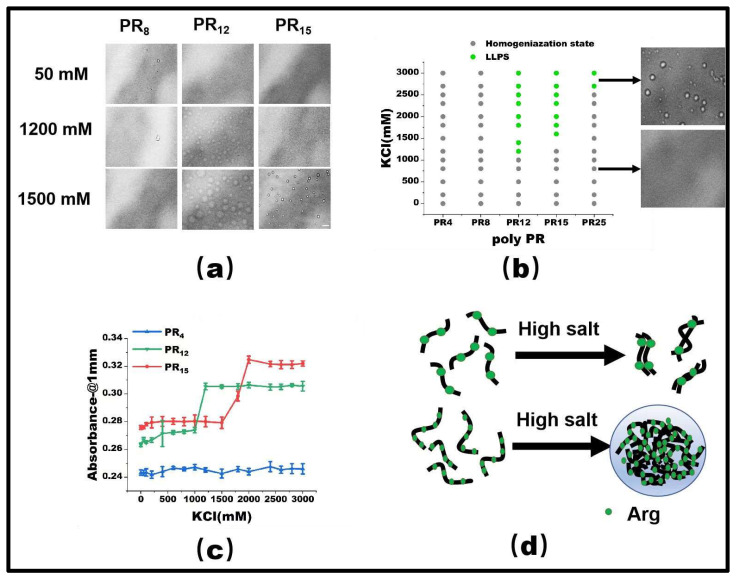
(**a**) The microscopic images for PR series at various KCl concentrations in 50 mM Tris-HCl (pH 7.2). The scale bar is 5 μm. (**b**) Phase diagram of liquid–liquid separation range of PR series repeat peptides at various KCl concentrations. The gray point indicates that there is no phase separation, while the green point denotes LLPS. The peptide concentrations are PR4 (625 μM), PR8 (312.5 μM), PR12 (208 μM), PR15 (166.6 μM), and PR25 (100 μM) in 50 mM Tris-HCl (pH 7.2) (**c**) The absorbance curve at 220 nm for PR4, PR12, and PR15 solution. (**d**) The schematic explanation for the LLPS: in the high salt state, the short peptide is saturated due to the few interaction sites, while in the high salt state, the long peptide is condensed due to the many interaction sites.

PR15 serves as a representative peptide for our LLPS investigation. Initially, at a 50 mM KCl concentration, PR15 remains in a homogeneous state. However, upon escalating the KCl concentration to 1500 mM, droplet formation became evident, hinting at its phase separation (Figure 1a). By mapping the phase diagram based on the observed microscopic patterns (Figure 1b), we noticed phase separation in PR12 and PR25. In contrast, the shorter PR4 and PR8 failed to exhibit any notable phase separation, regardless of being in low (50 mM) or high salt (2700 mM) environments. Specifically, PR12 demonstrated liquid–liquid separation at a salt concentration of 1200 mM (Figure 1a). Furthermore, using a UV spectrophotometer to examine the absorbance provided corroborative data; higher absorbance was observed in regions where liquid phase separation occurred at various salt concentrations (Figure 1c).

As observed before, the repetitive PR series peptides remain uniformly distributed in solution at low salt concentrations, while they undergo LLPS at high salt concentrations. This phenomenon can be ascribed to the synergetic effect of hydrophobic and electrostatic interactions between these peptides. In low salt conditions, the electrostatic repulsion between positively charged arginine units hinders the LLPS, despite the hydrophobic attraction between the peptides. In contrast, at high salt concentrations, the electrostatic repulsion between Arg-Arg pairs in the low salt state becomes a weak mutual attraction [2]. As the polymer length decreases, both PR8 and PR4 show LLPS behavior in KCl solutions, while remaining well-mixed homogeneous phases below the critical concentration. For example, PR4 and PR8 have fewer interacting sites than those longer chains, resulting in the phase separation behavior being unable to be realized (Figure 1d).

At low salt concentrations, poly PR itself is positively charged. Thus, the positively-charged arginines naturally repel each other through electrostatic interaction, consequently inhibiting the polymerization of peptides. In contrast, at the high salt concentration, arginine in poly PR lends it a hydrophilic character, which further promotes the LLPS. This interaction change is influenced by the repulsion between arginines in high salt conditions, which in turn amplifies its hydrophobic interactions [24]. This mechanism has been validated through both experimental data and all-atom molecular dynamic simulations [2].

**Figure 2:**
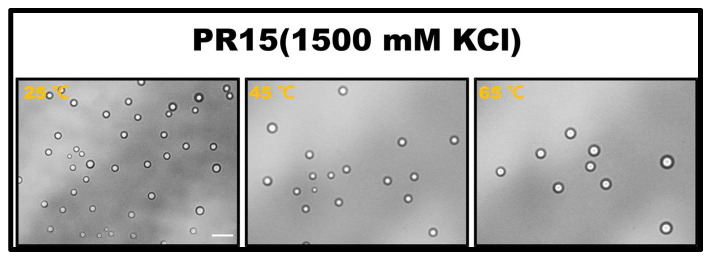
The microscopic images for PR15 at various temperatures in 50 mM Tris-HCl (pH 7.2). The scale bar is 5 μm.

The propensity for a short polypeptide to undergo LLPS at a diminished salt concentration might be attributed to the conformational entropy of the polypeptide chain. Specifically, the conformational entropy of a lengthy chain surpasses that of a shorter one, necessitating a more elevated salt concentration to amplify the attraction between R-groups and counterbalance the heightened conformational entropy. We also examined the impact of temperature on LLPS, with representative results illustrated in Figure 2. It is evident that an uptick in temperature obstructs the LLPS of PR15 due to the entropic cost. Increasing temperature does not mean promoting LLPS directly in the system. In this system, the increase in temperature changes the conformational entropy of peptides, which hinders the mutual condensation of peptides and inhibits the occurrence of LLPS [25,26]. In essence, the system’s free energy takes precedence in determining the LLPS of the peptide solution, being influenced by the number of active sites and the system’s conformational entropy. For exceptionally short peptides, like PR4 and PR8, LLPS is absent due to an insufficient count of active interchain sites. Conversely, for elongated peptides, the discrepancy in conformational energy prior to and following LLPS is substantial, thus necessitating the intensification of chain attraction by augmenting the salt concentration in the solution. The phase diagram in Figure 1b delineates a positive correlation between the requisite salt concentration and the peptide chain length.

### 2.2. LLPS of PR Peptide Series Regulated by Molecular Interaction Adjusting Agents

**Figure 3:**
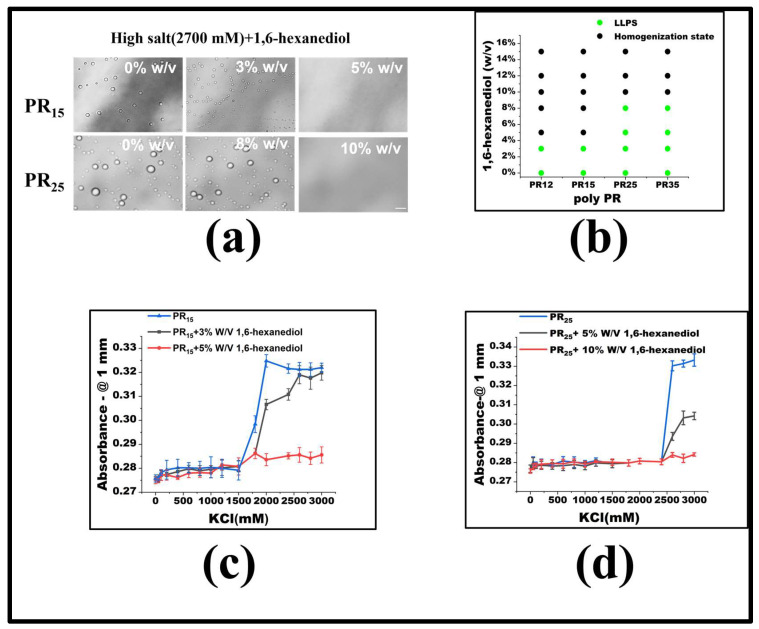
(**a**) Microscopic images of PR15 and PR25 with different concentrations of 1,6-hexanediol in KCl (2700 mM). All scales are 5 μm. (**b**) The phase diagram of PR polypeptides with 1,6-hexanediol. (**c**,**d**) The UV absorbance curves of PR15 and PR25 at different concentrations of 1,6-hexanediol.

For PR15, LLPS is triggered when the KCl concentration exceeds 1500 mM. To delve into the underlying forces driving the LLPS in the PR peptide series, we introduced molecular interaction modifiers at a set KCl concentration. Specifically, we employed 1,6-hexanediol, recognized as a hydrophobic destructive agent [2,27]. Maintaining a constant KCl concentration, we noted that by incrementally introducing 1,6-hexanediol, droplets present in high KCl concentrations began to dissipate (Figure 3a). Notably, once the concentration of 1,6-hexanediol reached 5%, PR15-induced droplets were entirely resolved. Previous studies indicate that 1,6-hexanediol can dissolve liquid-like protein clusters, yet not the solid-like protein formations [27]. Although the granular mechanism by which 1,6-hexanediol impacts liquid aggregates remains elusive, it is currently viewed as a hydrophobic agent that disrupts aggregates by negating the feeble attractions between peptides or proteins [2]. The mitigating influence of 1,6-hexanediol on PR peptide LLPS underscores the significance of hydrophobic interactions in the LLPS mechanism of PR peptides (Figure 3b).

To gauge the consistency of this observation across the PR peptide series, identical experiments were executed on PR12 and PR25. The outcomes revealed that 1,6-hexanediol had comparable effects on these peptides (Figure 3a,b). Ultraviolet spectrophotometry further corroborated this, indicating heightened absorbance both where LLPS occurred and where it did not (Figure 3c,d). Thus, incorporating 1,6-hexanediol into the KCl solution consistently reduces hydrophobicity, curbing peptide aggregation, and thereby impeding the LLPS.

Electrostatic interactions also significantly influence the LLPS of the PR peptide series. Their regulation can be achieved by blending two solutions possessing distinct dielectric properties. The composite solution’s dielectric constant is expressed as:

(1) $$\varepsilon =\varepsilon_{1}\phi_{1}+\varepsilon_{2}\phi_{2}$$

Here, $\varepsilon_{1}$ and $\varepsilon_{2}$ are the dielectric constants of solution 1 and 2 at equivalent temperature, while $\phi_{1}\;\mathrm{and}\;\phi_{2\;}$ denote their volume fractions, respectively. Notably, the dielectric constant of ethyl alcohol approximates 24, indicating an enhancement of electrostatic interaction. In contrast, a 1 M 6-Aminocaproic acid (6-A) solution boasts a dielectric constant of 157.5, exceeding water’s 80, suggesting its potential to mitigate the electrostatic interactions of charged biomolecules in the solution [28,29,30].

**Figure 4:**
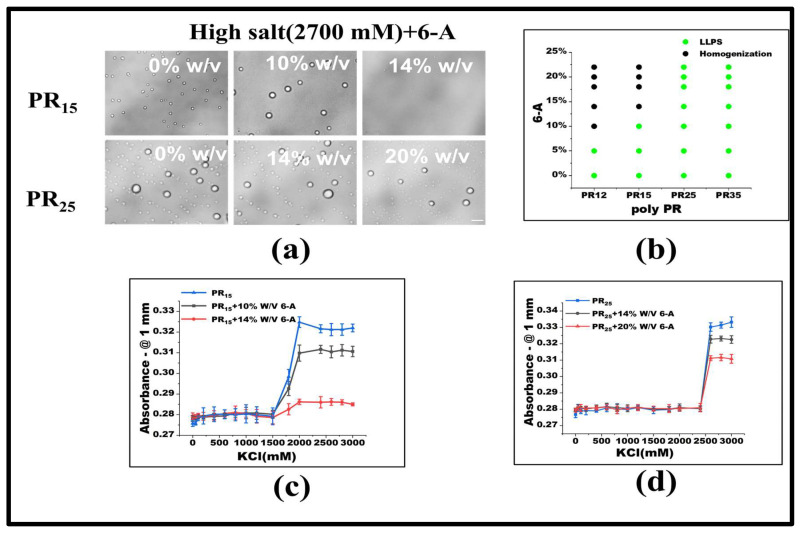
(**a**) Microscopic images of PR15 and PR25 with different concentrations of 6-A in KCl (2700 mM). All scales are 5 μm. (**b**) The phase diagram of PR polypeptides with 6-A. (**c**,**d**) The UV absorbance curves of PR15 and PR25 at different concentrations of 6-A.

Under consistent KCl concentrations, various 6-A concentrations were added. For PR15, LLPS ceases when 6-A concentration reaches 14% *w*/*v*. Similarly, for PR12, LLPS halts at a 10% *w*/*v* concentration of 6-A (Figure 4a,b). In other systems, like PR25, the influence of 6-A also notably diminishes LLPS to some extent (Figure 4b). Absorbance variations in PR15 and PR25 under 6-A’s influence further underscore this (Figure 4c,d).

Under low salt conditions, Arg-Arg interactions manifest as electrostatic repulsions. Yet, in high salt environments, these evolve into weakly attractive π–π interactions. This shift emerges when the repulsion between positive guanidines is overshadowed, allowing sp2 hybrid planar guanidines to connect via their π orbitals [24,31,32]. The influence of 6-A on PR12, PR15, and PR25 arises from its effect on the high-salt-concentration LLPS, which primarily hinges on the non-ionic interaction between arginine units stemming from their inherent electrostatic repulsion. Thus, introducing 6-A attenuates this arginine–to–arginine electrostatic repulsion, weakening the non-ionic interactions at elevated salt concentrations, and ultimately suppressing LLPS.

In exploring electrostatic interactions, prior research has identified the fact that 6-A can attenuate these interactions, while ethyl alcohol amplifies them. This adjustment is primarily due to the reduction in the dielectric constant by these agents [28,29,30]. Based on this understanding, we theorized that ethyl alcohol might modulate the LLPS of PR polypeptides, potentially promoting LLPS at high salt concentrations.

**Figure 5:**
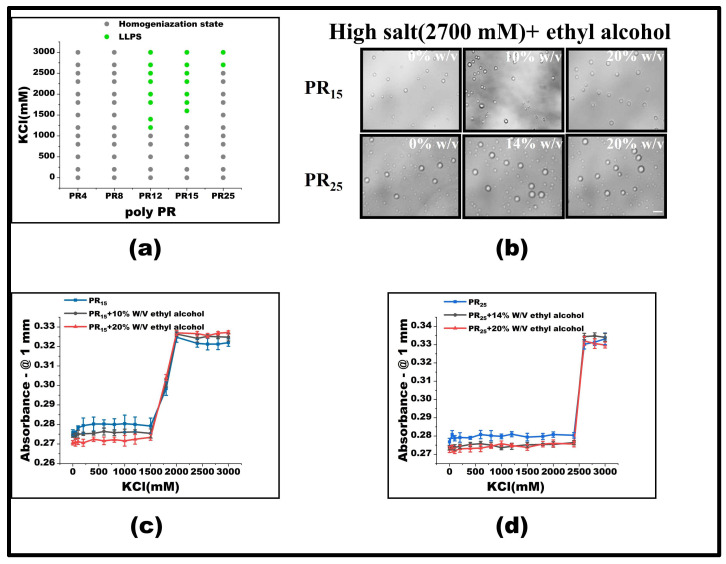
(**a**) Phase diagram of liquid–liquid separation range of PR series repeat peptides at various KCl concentrations. The gray point indicates that there is no phase separation, while the green point denotes LLPS. The mass concentration of ethyl alcohol added to the same polypeptide is 0%, 10%, and 20%, in turn. (**b**) Microscopic images of PR15 and PR25 with different concentrations of alcohol in KCl (2700 mM). All scales are 5 μm. (**c**,**d**) The UV absorbance curves of PR15 and PR25 at different concentrations of alcohol.

To test this hypothesis, we introduced varying concentrations of ethyl alcohol at the pivotal KCl concentration where LLPS was absent, focusing on observing the behavior of PR15. Our findings revealed that, with a 10% *w*/*v* inclusion of ethyl alcohol, PR15’s LLPS was enhanced, broadening the LLPS phase diagram’s range (Figure 5a). Yet, at KCl concentrations already inducing LLPS, ethyl alcohol’s modulatory effect was negligible (Figure 5b–d). Interestingly, PR25 did not respond effectively to ethyl alcohol at critical KCl concentrations where LLPS was absent (Figure 5a–d).

**Figure 6:**
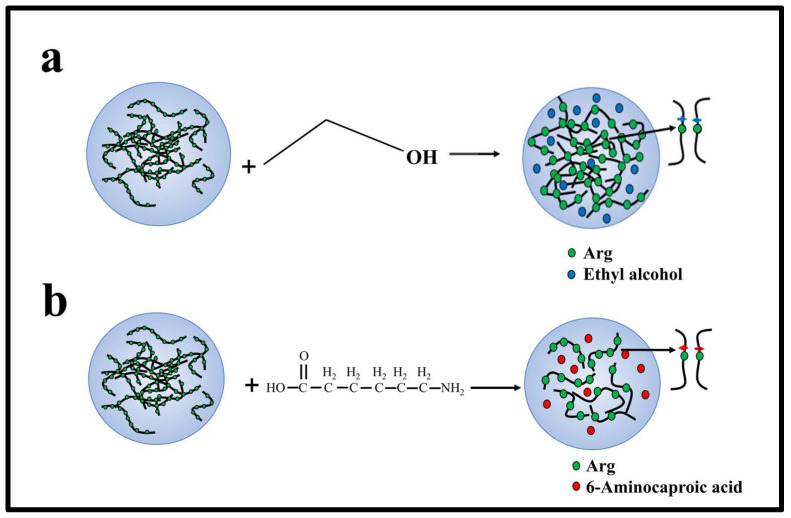
Mechanism of interaction between charged amino acids, ethyl alcohol, and 6-A of PR polypeptides in solution. (**a**) The interaction between ethyl alcohol and Arg in solution. (**b**) The interaction between 6-A and Arg in solution.

When LLPS is yet to occur, PR12 and PR15’s response to ethyl alcohol can be attributed to the agent enhancing the non-ionic interaction between arginine residues, thus fostering phase separation. However, this modulatory effect becomes ineffectual once phase separation has been initiated. We postulate that, in high salt concentrations, hydrophobic interactions predominantly govern the phase separation, whereas the addition of ethyl alcohol predominantly influences electrostatic interactions. Essentially, once in a phase-separated system, the arginine–arginine interaction reaches saturation, making further modulation by alcohol ineffective (Figure 6).

In summary, at low salt concentrations, the PR polypeptide does not undergo LLPS, mainly due to prevailing electrostatic repulsion. In contrast, at higher salt states, its interactive behavior transitions from electrostatic repulsion to mutual attraction.

Based on our experimental findings, it is evident that both short- and long-peptide LLPS are inhibited when the dielectric constant is increased using 6-A, leading to diminished electrostatic forces. Introducing anhydrous ethanol into the system enhances the electrostatic force, but only by approximately 15%, given its relatively minor volume (less than 20%). This modulation is more pronounced for short peptide chains, while longer peptide chains display a subdued response. This disparity arises because the dominant factor influencing the LLPS of long peptide chains is their conformational entropy, rendering the interchain electrostatic energy less consequential.

### 2.3. The Synergetic Regulation of Liquid–Liquid Separation of PR15 in Two Ways

From our experiments, we established that while 1,6-hexanediol inhibits the LLPS of the PR polypeptide, ethyl alcohol tends to promote it. This led us to investigate whether a reversible regulation of LLPS could be achieved using these compounds. We zeroed in on the PR15 and PR25 polypeptides for this exploration.

**Figure 7:**
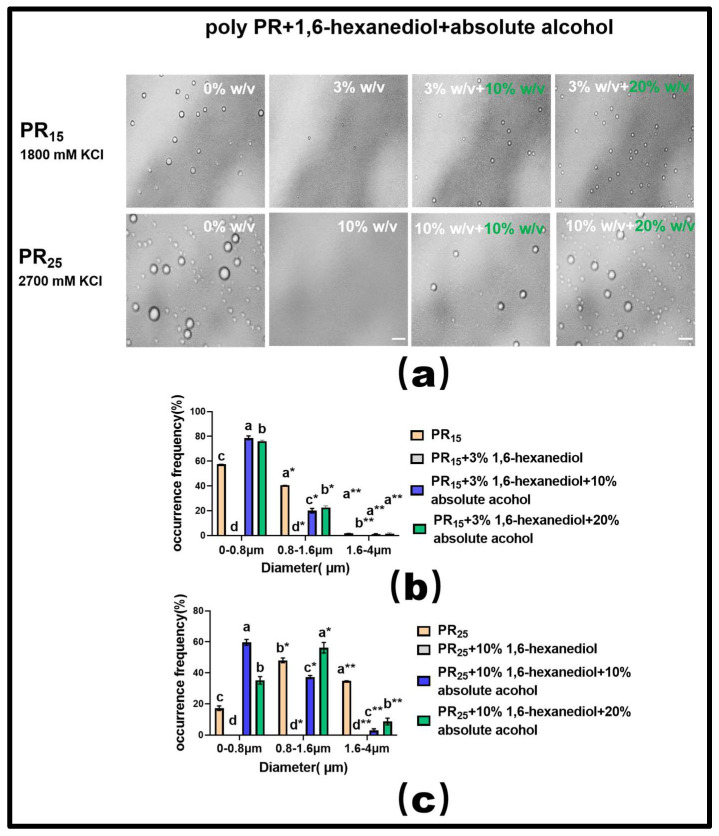
(**a**) Microscopic Images of PR15 with different concentrations of 1,6-hexanediol and ethyl alcohol in KCl (1800 mM) and PR25 with different concentrations of 1,6-hexanediol and ethyl alcohol in KCl (2700 mM). All scales are 5 μm. (**b**,**c**) Distributions of the diameter of droplets of PR15 and PR25 under different solution conditions. The letters a–d in subfigures (**b**,**c**) represents a significant difference of diameters. The symbol * and ** indicates different groups of diameters.

For PR15, at a KCl concentration of 1800 mM, introducing a 3% mass concentration of 1,6-hexanediol effectively dissolved the solution’s droplets (Figure 7a). Building on this, adding varying concentrations of ethyl alcohol showed that a 20% concentration exhibited a more-pronounced LLPS restoration than a 10% concentration (Figure 7a). However, it is noteworthy that even with this restoration, the droplets did not revert to their original state seen before the addition of 1,6-hexanediol. This indirectly underscores the hydrophobic interaction’s centrality in driving the LLPS of the PR polypeptide under high salt conditions (Figure 7a).

For PR25, at a KCl concentration of 2700 mM, adding 10% mass concentration of 1,6-hexanediol also almost completely dissolved the droplets (Figure 7a). When different concentrations of ethyl alcohol were subsequently introduced, the 20% concentration, similar to PR15, more effectively restored the LLPS compared to the 10% concentration (Figure 7a).

In summary, these experiments suggest that the bidirectional regulation of PR polypeptide LLPS can be realized through the combined effects of hydrophobic and electrostatic interactions. Additionally, our analysis of the droplet diameter’s frequency distribution in the solution corroborated these findings (Figure 7b,c).

Moreover, extensive experimental research in phase separation has consistently highlighted the fact that the molecular crowding effect facilitates the onset of the LLPS phenomenon [33,34]. In line with this understanding, we employed a molecular crowding agent, PEG1000, in conjunction with the hydrophobic disruptor, 1,6-hexanediol, to modulate the LLPS of PR polypeptides in our experiments.

**Figure 8:**
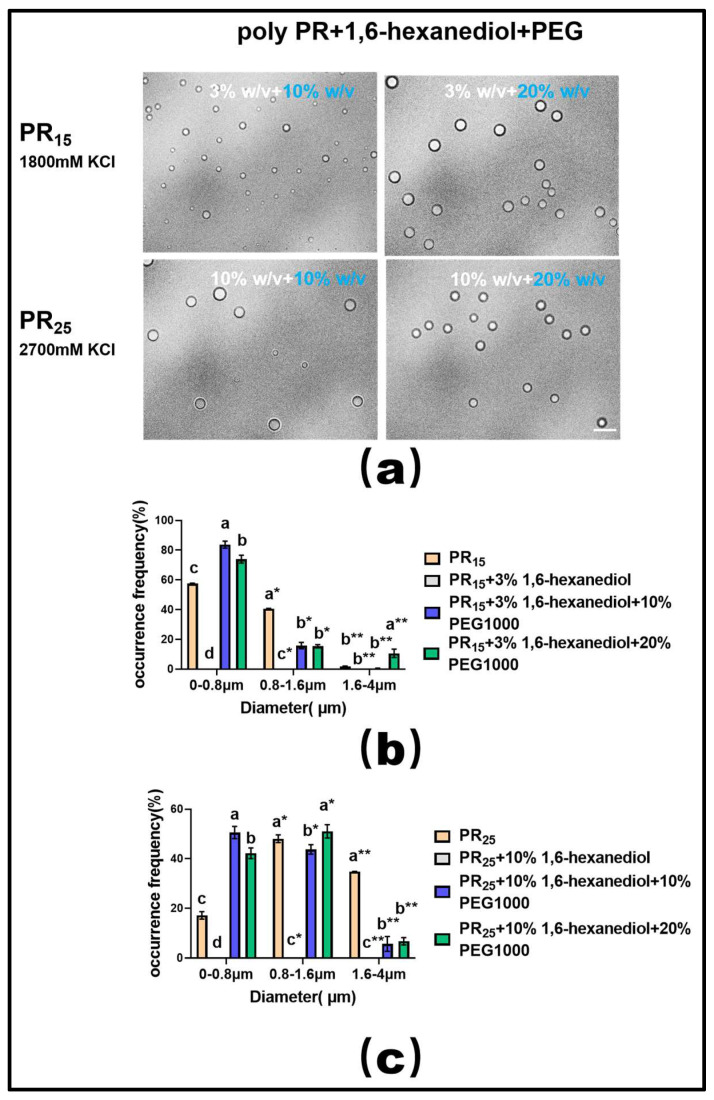
(**a**) Microscopic Images of PR15 with different concentrations of 1,6-hexanediol and PEG1000 in KCl (1800 mM) and PR25 with different concentrations of 1,6-hexanediol and PEG1000 in KCl (2700 mM). All scales are 5 μm. (**b**,**c**) Distributions of the diameter of droplets of PR15 and PR25 under different solution conditions. The letters a–d in subfigures (**b**,**c**) represents a significant difference of diameters. The symbol * and ** indicates different groups of diameters.

Upon utilizing a suitable concentration of 1,6-hexanediol to dissolve the PR polypeptide droplets, we observed that introducing varying mass concentrations of PEG1000 effectively restored droplet formation in both PR15 and PR25 solutions (Figure 8a). Furthermore, data on droplet diameter frequency distribution revealed that this recovery effect, driven by PEG1000, surpassed the combined influence of ethyl alcohol and 1,6-hexanediol (Figure 8b,c).

## 3. Materials and Methods

### 3.1. Reagent Preparation

All reagents and chemicals were purchased with the highest purity available. Ethyl alcohol, 6-Aminocaproic acid, and 1,6-hexanediol were obtained from Sigma Aldrich (St. Louis, MO, USA) and a 40% (*w*/*v*) stock solution was prepared in 50 mM Tris-HCl (pH 7.2). The Pro-Arg repeats were obtained from Jiangsu Ji Tai Peptide Industry Science and Technology Co, Ltd. (Suzhou, China) as lyophilized powder and dissolved in 50 mM Tris-HCl (pH 7.2). PEG1000 was purchased from Sigma Aldrich and a 50% (*w*/*v*) stock solution was prepared in 50 mM Tris-HCl (pH 7.2). Purified water was obtained from Sichuan Youpu Ultrapure Technology (Chengdu, China). The original mass concentration of ethyl alcohol in the experimental process was 99.7%, and the concentration was diluted to 10%, 14% and 20% in the subsequent experiments with ethyl alcohol.

### 3.2. Observation and Measurement

We used an inverted optical microscope (Nikon, Tokyo, Japan) to observe droplets from LLPS. The images were captured at 40× or 60× (oil immersion) magnification, and the diameter of LLPS droplets was measured using the Image J software (2023 version) equipped with the microscope. The INSTEC heating and freezing microscope stage (Shanghai, China) was used to control the temperature.

Appropriate amounts of salt, water, and additives were added to the peptide stock solutions and mixed by pipetting to induce LLPS in the solution. The buffer of 50 mM Tris-HCl (pH 7.2) was used in all cases. The samples were prepared in tubes and imaged within 1–5 min to limit any aging effects. Phase diagrams of the solution were constructed by observing the droplets in the solution at various concentrations of additives.

A Nikon ECLIPSE Ti-S inverted phase-contrast microscope (Nikon, Tokyo, Japan) was used for the measurement. We used a 100× oil lens (Plan fluor 100×/1.30 Oil) and an attached CCD camera to obtain images of solution on slides at the temperature-controlled stage.

Measurement of UV absorbance: specific quantities of PR repeat peptides of varying polymerization degrees were dissolved in Tris-HCl buffer and preserved at 4 °C. Concentrations were adjusted to ensure consistent amino acid numbers across varying PR peptide polymerizations. The diluted peptides were then treated with KCl at concentrations ranging from 50 mM to 3 M. Tris-HCl buffer devoid of KCl was used as blank control. After a 5 min reaction at 25 °C, 2 µL of the prepared solution was loaded onto the pedestal of the Q5000 ultra-micro ultraviolet spectrophotometer (Quawell, San Jose, CA, USA) for absorbance measurement at 220 nm. We chose 1 mm for our measurement, conveniently, since it is the default length of the spectrophotometer. Most amino acids, including arginine and proline, have only one absorption peak in the ultraviolet region, and the absorption peak is mostly concentrated in 200–220 nm. There is no light absorption in the ultraviolet region of 230 nm, so we used the 220 nm absorption wavelength in the measurement process [35,36,37]. In the present study, we used the turbidity not quantitatively, but only for verification of LLPS, since the direct images were presented. The wavelength of 220 nm was chosen only for convenience in the spectroscopy, and does not influence our conclusion, although it might affect some absorbance of the components in the solution.

## 4. Conclusions

In our exploration, we delved into the LLPS behavior of repetitive proline-arginine (PR) polypeptides in solutions across varied salt concentrations and additives. Key findings include:

LLPS Onset: LLPS in PR polypeptides manifested exclusively at KCl concentrations surpassing 1200 mM. Intriguingly, as the sequence length augmented, so did the requisite concentration. Notably, PR polypeptides with fewer than 12 repeat sequences did not exhibit LLPS, regardless of the KCl concentration.

Deciphering Molecular Drivers: To elucidate the molecular impetus steering LLPS in PR polypeptides, we introduced both hydrophobic shielding agents and reagents modulating electrostatic interactions to solutions containing elevated KCl concentrations. Our data elucidated the fact that introducing the hydrophobic shielding agent, 1,6-hexanediol, to PR polypeptides experiencing LLPS effectively neutralized the occurrence. Meanwhile, the integration of 6-aminocaproic acid impeded LLPS, and, conversely, ethyl alcohol bolstered LLPS in PR polypeptides.

Controlling LLPS Reversibility: Harnessing 1,6-hexanediol, ethyl alcohol, and PEG, we manipulated the reversibility of PR polypeptide LLPS. Specifically, while 1,6-hexanediol’s inhibitory action curtailed the LLPS of PR polypeptides, judiciously administering ethyl alcohol facilitated partial recuperation. Furthermore, post 1,6-hexanediol treatment, the judicious addition of PEG also resurrected the LLPS phenomenon.

In summary, our insights, stemming from these rigorous experiments, hold promise in potentially enlightening therapeutic avenues and drug development endeavors targeting phase-separation-associated ailments.

## Data Availability

Not applicable.

## Sample Availability

Samples of the compounds are available from the authors.
